# The effect and application of adiponectin in hepatic fibrosis

**DOI:** 10.1093/gastro/goae108

**Published:** 2024-12-30

**Authors:** Taoran Chen, Wenjing Yang, Rongrong Dong, Han Yao, Miao Sun, Jiaxin Wang, Qi Zhou, Jiancheng Xu

**Affiliations:** Department of Laboratory Medicine, First Hospital of Jilin University, Changchun, Jilin, P. R. China; Department of Laboratory Medicine, First Hospital of Jilin University, Changchun, Jilin, P. R. China; Department of Laboratory Medicine, First Hospital of Jilin University, Changchun, Jilin, P. R. China; Department of Laboratory Medicine, First Hospital of Jilin University, Changchun, Jilin, P. R. China; Department of Laboratory Medicine, First Hospital of Jilin University, Changchun, Jilin, P. R. China; Department of Laboratory Medicine, First Hospital of Jilin University, Changchun, Jilin, P. R. China; Department of Pediatrics, First Hospital of Jilin University, Changchun, Jilin, P. R. China; Department of Laboratory Medicine, First Hospital of Jilin University, Changchun, Jilin, P. R. China

**Keywords:** adiponectin, hepatic fibrosis, cirrhosis, endoplasmic reticulum stress, hepatic stellate cell

## Abstract

Hepatic fibrosis, a degenerative liver lesion, significantly contributes to the deterioration and mortality among patients with chronic liver diseases. The condition arises from various factors including toxins, such as alcohol, infections like different types of viral hepatitis, and metabolic diseases. Currently, there are no effective treatments available for liver fibrosis. Recent research has shown that adiponectin (ADPN) exhibits inhibitory effects on hepatic fibrosis. ADPN, an adipocytokine secreted by mature adipocytes, features receptors that are widely distributed across multiple tissues, especially the liver. In the liver, direct effects of ADPN on liver fibrosis include reducing inflammation and regulating hepatic stellate cell proliferation and migration. And its indirect effects include alleviating hepatic endoplasmic reticulum stress and reducing inflammation in hepatic lobules, thereby mitigating hepatic fibrosis. This review aims to elucidate the regulatory role of ADPN in liver fibrosis, explore how ADPN and its receptors alleviate endoplasmic reticulum stress, summarize ADPN detection methods, and discuss its potential as a novel marker and therapeutic agent in combating hepatic fibrosis.

## Introduction 

Cirrhosis ranks as the eleventh leading cause of death globally. Deaths attributed to cirrhosis increased from 899,000 annually in 1990 to 1.32 million in 2017. The Global Burden of Disease study estimates that cirrhosis accounts for approximately 2 million deaths annually, primarily due to hepatic fibrosis [1]. Hepatic fibrosis, a chronic liver condition, results from various factors, including toxins (e.g. alcohol), viral infections (e.g. hepatitis virus), and metabolic disorders [2]. This condition is characterized as a degenerative liver lesion [3]. Post-liver injury, interactions among damaged cells, inflammatory cells, and hepatic myofibroblasts lead to hepatic fibrosis development. Such progression may lead to cirrhosis, liver failure, and eventually hepatocellular carcinoma. At present, the main treatment of hepatic fibrosis is to eliminate the causes of chronic liver injury. Direct and effective therapeutic interventions remain elusive. Therefore, elucidating the transforming process of liver fibrosis can aid in the study of anti-hepatic fibrosis therapy.

**Figure 1.**
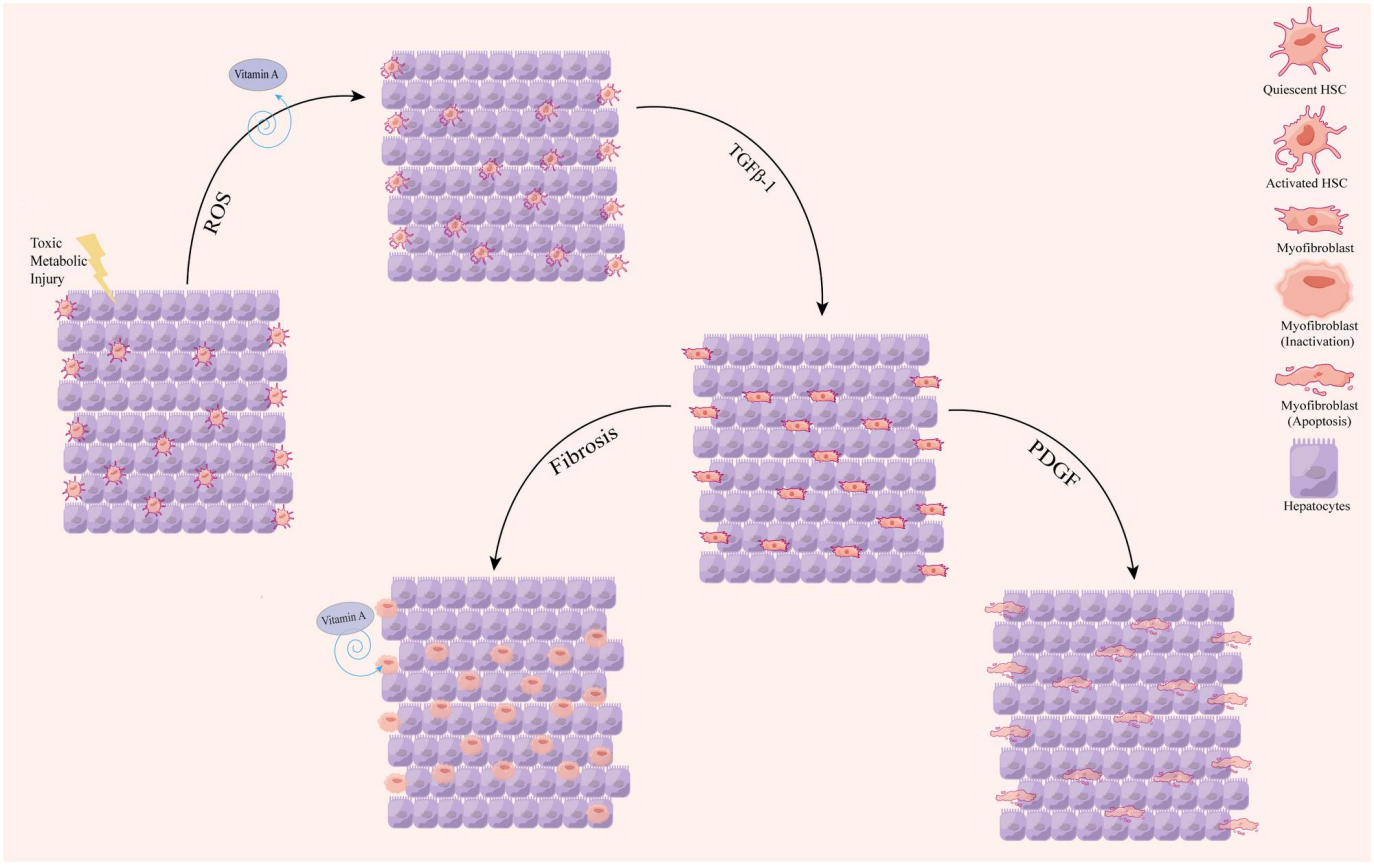
The process of HSC development. The figure showed that hepatocyte injury triggers an inflammatory response that releases ROS and transforming growth factor β1 (TGFβ-1) and activates qHSC-aHSC myofibroblasts. aHSC/myofibroblasts proliferate in response to a variety of cytokines (PDGF), secrete type I collagen, and fibrogenize the liver. One key feature of qHSC is the expression of vitamin A, which is gradually down-regulated during the transition to aHSC and restored after aHSC inactivation. HSC = hepatic stellate cells, ROS = reactive oxygen species, TGFβ-1 = transforming growth factor β1, qHSC = quiescent HSCs, aHSC = activated form of HSCs.

Hepatic fibrosis morphology is characterized by an excessive accumulation of the extracellular matrix, predominantly composed of fibrillar collagen [4]. Hepatic stellate cells (HSCs), a type of fibroblast, become activated following liver injury, contributing to connective tissue formation and extracellular matrix accumulation. In normal liver tissue, quiescent HSCs (qHSCs) constitute 15% of the cell population, maintaining their quiescent state by relying on stored vitamin A [5]. In the early stages of liver fibrosis, reactive oxygen species (ROS) activate transforming them into their activated form (aHSCs) [6], which are the primary drivers of liver fibrosis (Figure 1). aHSCs can transform into myofibroblasts, secreting significant quantities of collagen and extracellular matrix, thereby accelerating hepatic fibrosis progression [7]. Research has demonstrated that liver fibrosis may be reversible in its early stages if aHSCs undergo apoptosis or revert to a dormant state, known as the regression phase [8]. Consequently, identifying the cellular and molecular pathways involved in HSC activation is crucial for developing effective treatments for liver fibrosis.

**Figure 2.**
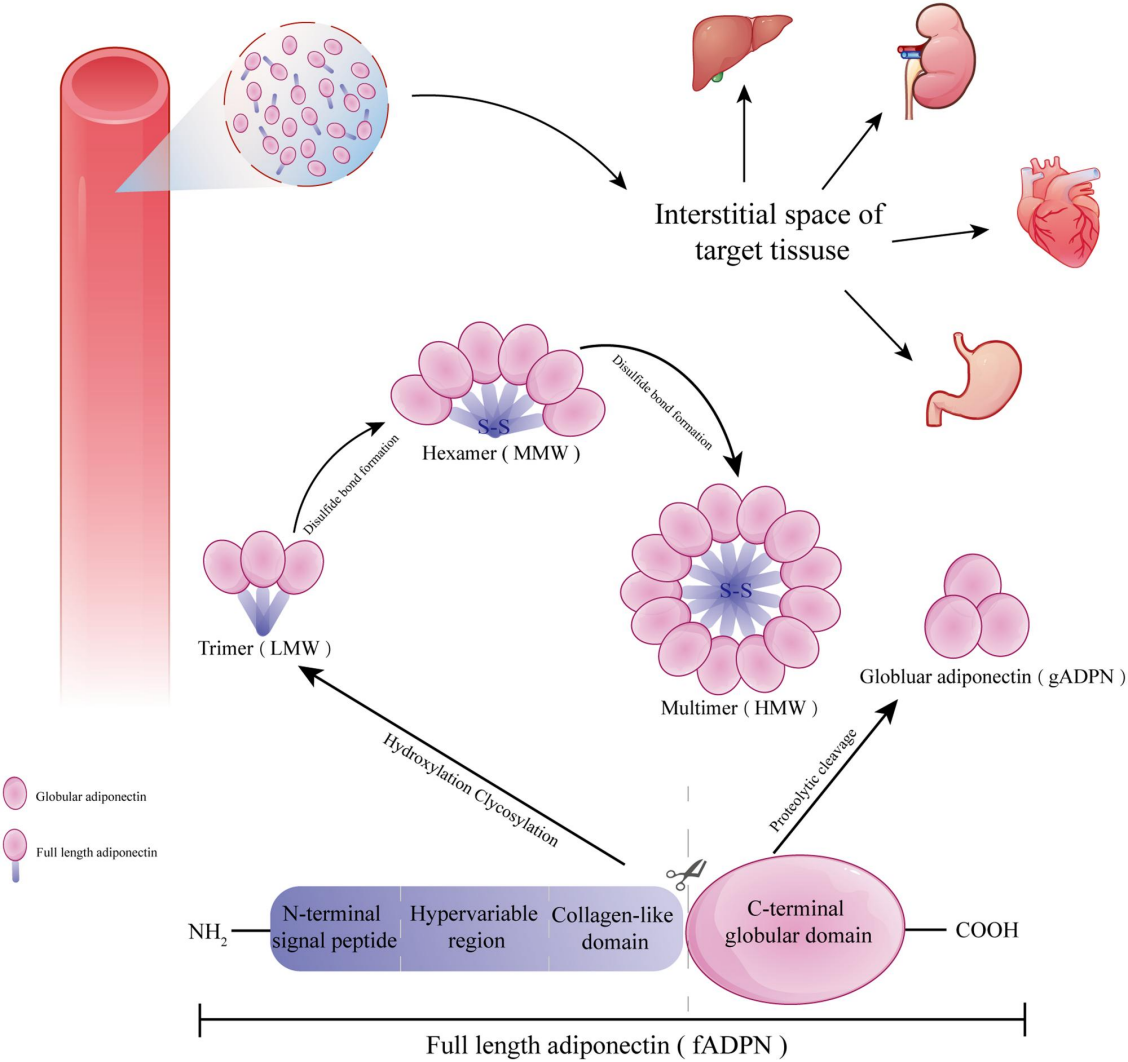
The structure of ADPN. Adiponectin exists as a full-length plasma protein of 30 kDa, circulating in serum as three major isoforms: trimer, hexamer, and high-molecular-weight multimer. Extravasation of ADPN from circulation to target tissues. ADPN = adiponectin.

Adiponectin (ADPN), a 30-kDa plasma protein, is abundantly secreted by adipose tissue and constitutes about 0.01%–0.05% of total plasma proteins [9, 10]. ADPN comprises four major structural domains: the N-terminal signal peptide, the hypervariable region, the collagen-like domain, and the C-terminal globular domain [11]. Studies have shown that full-length ADPN exists in serum predominantly in three forms: trimeric, hexameric, and high-molecular-weight multimers [12, 13]. Once formed, these polymers display unique biochemical properties that do not interconvert during their lifecycle [14]. The isolated globular domain of ADPN retains biological activity, albeit at significantly lower levels compared to other ADPN multimers [15]. Globular ADPN, a form present in the ADPN cycle, has been demonstrated to exert beneficial effects on metabolic processes, in addition to enhancing macrophage activity [16]. The different forms of ADPN are shown in Figure 2. Most circulating ADPN originates from adipocytes [17], which predominantly secrete adipokines that induce pro-inflammatory effects, like tumor necrosis factor alpha (TNF-α). Unlike other adipokines, ADPN exhibits anti-inflammatory, antidiabetic, and cardioprotective properties and plays a significant role in cancer [18–20]. The metabolic impacts of ADPN on skeletal muscle and liver have been extensively documented [21, 22]. Additionally, the study confirmed ADPN’s similar beneficial effects in the heart, enhancing glucose uptake and fatty acid metabolism [23].

The anti-fibrotic effects of ADPN in the liver from its ability to modulate HSC activation and reduce inflammation [24, 25]. Chronic inflammation triggers hepatocyte necrosis and apoptosis, leading to the release of inflammatory mediators by Kupffer cells through the TLR-4 signaling pathway. These inflammatory mediators activate HSCs, which subsequently phagocytize the dead hepatocytes [26]. ADPN inhibits the TLR-4 signaling pathway in Kupffer cells, thus preventing the proliferation and migration of HSCs [25].

This review explored alterations in ADPN levels in liver fibrosis and its regulatory functions, elucidates how ADPN and its receptors reduce endoplasmic reticulum stress (ERS) in hepatocytes, surveys current ADPN detection methods, and evaluates the potential of ADPN as a promising biomarker for early diagnosis of liver fibrosis and as a therapeutic agent for liver fibrosis treatment.

## Biliary atresia caused by liver fibrosis may increase ADPN

Biliary atresia represents a common complication associated with hepatic fibrosis. Patients with biliary obstruction-type disease often exhibit more severe hepatic fibrosis than those with other types of biliary complications [27]. Earlier research indicated significant elevations in ADPN levels in patients with biliary diseases linked to hepatic fibrosis, suggesting biliary excretion plays a role in ADPN clearance [28]. ADPN levels passively increase due to reduced excretion in hepatic fibrosis. Animal experiments involving mice subjected to bile duct ligation, thereby obstructing bile excretion and inducing cholestasis, revealed a 2- to 3-fold increase in serum ADPN levels 6-h post-surgery [28]. Serum ADPN levels exhibited a linear increase 3 days following surgery. The study assessed ADPN gene expression in the mouse liver pre- and post-surgery using RT-PCR, with results indicating no increase in ADPN gene expression, suggesting choledochotomy does not promote ADPN expression from the mouse liver [28]. Furthermore, Tacke *et al.* observed ADPN concentrations in the bile of patients with severe biliary obstruction to be 100-fold higher than those in serum, underscoring the significance of bile secretion as a vital pathway for ADPN clearance, with biliary obstruction diminishing ADPN excretion and consequently increasing its accumulation in the body [28].

## ADPN can alleviate hepatic fibrosis

### Anti-inflammation effect of ADPN

Inflammation promotes liver fibrosis development [8]. Following liver injury, inflammatory cells are recruited to participate in the clearance of damaged hepatocytes, contributing to fibrosis formation. ADPN has demonstrated potent anti-inflammatory properties, exhibiting both rapid and transient effects in animal and human studies [29]. ADPN mediates anti-inflammatory effects across various diseases, including type 2 diabetes mellitus, hepatic fibrosis, and cardiovascular disease [30, 31], primarily by modulating macrophage differentiation [32]. This modulation involves inhibiting the activation of pro-inflammatory M1 phenotype macrophages and promoting a shift toward the anti-inflammatory M2 phenotype. Increased M1 markers (TNF-α, IL-6, and monocyte chemotactic protein-1) and decreased M2 markers (arginase 1 and IL-10) in ADPN knockout mice indicate the direct role of ADPN in macrophage polarization [33] and its indirect effects through monocyte-mediated inflammatory mediators [34]. ADPN not only diminishes the expression of inflammation-associated receptors but also curtails the production of inflammatory chemokines while promoting IL-10, an anti-inflammatory cytokine, in macrophages [35]. Additionally, ADPN attenuates the infiltration of inflammatory cells and overexpression of inflammatory genes induced by fibrotic growth factors [36–38].

### ADPN binds to receptors to regulate liver fibrosis

ADPN modulates liver function through the binding and activation of membrane receptors [39, 40], notably AdipoR1 and AdipoR2. AdipoR1 is predominantly expressed in muscle tissues, whereas AdipoR2 has its primary expression in the liver. Binding of ADPN to AdipoR2 alleviates hepatic fibrosis by activating downstream signaling pathways and attenuating hepatic inflammation [41]. Moreover, ADPN inhibits hepatic fibrosis by downregulating HSC activation markers, including alpha-smooth muscle actin (α-SMA), transforming growth factor β1 (TGFβ-1), and pro-fibrotic genes [42]. A study demonstrated that AdipoR1 knockout in mice had no significant impact on hepatic fibrosis progression, whereas AdipoR2 knockout exhibited a marked increase in hepatic fibrosis, highlighting the critical role of AdipoR2 in fibrosis development [42]. Reduced AdipoR expression diminished ADPN-AdipoR signaling, while enhanced AdipoR expression reinstated ADPN’s anti-fibrotic effects. Loss of ADPN signaling intensified fibrosis both *in vivo* and *in vitro* [43]. Animal studies further revealed that AdipoR agonist treatment activated adenosine monophosphate-activated protein kinase (AMPK) and peroxisome proliferator-activated receptor alpha (PPARα) signaling, significantly improving lipid metabolism and hepatic synthetic functions [44].

### ADPN inhibits HSC proliferation

HSC constitutes the primary source of pro-fibroblasts in the liver. Upon external stimulation, HSC can differentiate into myofibroblasts, proliferating in large numbers, migrating to the liver injury sites, and secreting excessive amounts of extracellular matrix, thereby inducing fibrosis [45].

AMPK acts as a pivotal regulator of the metabolic system, mitigating lipid oversynthesis and enhancing liver function through the inhibition of acetyl coenzyme A carboxylase (ACC1 and ACC2) phosphorylation [46]. ADPN, particularly high molecular weight ADPN, has been demonstrated to suppress downstream protein kinase B (AKT) signaling pathway via activating AMPK, a critical pathway in regulating HSC proliferation [47]. Studies have indicated that AMPK suppresses ROS production by upregulating antioxidant gene expression and inhibiting AKT activation, thereby mitigating HSC proliferation and hepatic fibrosis [48, 49].

### ADPN inhibits HSC migration

ADPN not only inhibited HSC proliferation but also suppressed HSC migration. In the context of hepatic fibrosis, the body commonly secretes tissue inhibitor of metalloproteinase 1 (TIMP-1), which has been found to promote the activation of survival signaling and NF-κB (nuclear factor kappa beta) pathways in HSCs [50], consequently downregulating the expression of pro-apoptotic pathways and maintaining the activity of aHSCs [51–53]. A study showed that in the presence of ADPN, TIMP-1 levels are unexpectedly elevated but that this in fact may assist in limiting fibrosis by reducing HSC invasion and migration through the extracellular matrix [25].

Moreover, a study shows that (1) despite well-described anti-fibrotic effects, ADPN can promote TIMP-1 expression *in vitro*, *in vivo*, and in patients; (2) this effect is mediated by ADPN-induced AMPK signaling, and (3) ADPN-stimulated TIMP-1 impairs HSC motility through binding to the CD63/β1-integrin complex to inhibit focal adhesion kinase (FAK) phosphorylation. Furthermore, a related study showed that CD63 interacts with β1-integrin, which in turn interacts with FAK, and observed with blocking antibodies to CD63 or TIMP-1 or with CD63 shRNA that FAK phosphorylation was increased compared with ADPN treatment alone, suggesting that reduced HSC migration in the presence of ADPN is modulated by FAK [25]. In conclusion, these results illustrate a novel pathway of ADPN–TIMP-1-induced inhibition of HSC migration.

### ADPN promotes apoptosis of HSCs

Previous research has demonstrated that ADPN plays a regulatory role in apoptosis [54]. Within the liver, apoptosis of aHSCs serves as a vital mechanism for preserving circulatory homeostasis and facilitating the spontaneous resolution of liver fibrosis [8, 51, 55, 56]. Apoptosis in aHSCs encompasses mechanisms, such as activation of death receptor-mediated pathways (e.g. FAS, TRAIL), activation of caspases (caspase-3, caspase-8), upregulation of pro-apoptotic proteins (p53, BAX), and activation of hepatic natural killer (NK) cells and T cells [8, 57–59]. Furthermore, ADPN has been documented to mitigate liver fibrosis by modulating the p53 pathway, reverting activated HSCs to qHSCs, and inducing aHSC senescence [31]. It has been demonstrated that ADPN is observed to induce apoptosis in activated HSCs in both *in vivo* and *in vitro* studies; however, the precise mechanism remains elusive and warrants further exploration [53].

### ADPN relieves ERS

The liver is central to systemic lipid metabolism, regulating lipid synthesis, oxidation, transport, and excretion. The hepatocyte endoplasmic reticulum, a critical organelle, is involved in key processes, including secretion, transmembrane protein folding, ion homeostasis, and lipid biosynthesis, which are crucial for hepatic metabolism. However, external factors can disrupt endoplasmic reticulum (ER) function, contributing to the development of liver fibrosis [60]. Numerous studies have confirmed the crucial role of ER in regulating liver fibrosis development, with abnormalities in its internal environment observed in many cirrhotic patients [44, 61, 62]. ERS often results in hepatocyte death. Research indicates that ADPN can mitigate ERS and the resulting inflammatory responses in chronic liver diseases, such as cirrhosis and liver injury [44, 63].

### Mechanisms of ERS affecting hepatic fibrosis

The endoplasmic reticulum is responsible for a range of critical biological functions, including the co-translational folding of proteins within the cytosol [64]. Both physiological and pathological stimuli, such as B cell maturation, glucose-induced insulin secretion, nutrient deprivation, oxidative stress, hypoxia, and mutations in ER-associated genes, can disrupt ER homeostasis, resulting in ER stress [65]. In response to ER stress, eukaryotic cells have adapted the ER-nucleus signaling system, commonly known as the unfolded protein response (UPR), to resolve ER stress or execute cell death. Under conditions of mild to moderate ER stress, the UPR is activated to eliminate unfolded or misfolded proteins and restore ER homeostasis. However, upon severe or persistent ER stress, UPR is hyperactivated, leading to the activation of intrinsic apoptotic machinery [66].

The activation of the UPR involves three key proteins localized to the ER: inositol-requiring enzyme 1-α (IRE1-α), PKR-like endoplasmic reticulum kinase (PERK), and activating transcription factor 6α (ATF6α) [67]. It is currently thought that these three proteins remain in an inactive state through their association with the ER chaperone glucose-regulated protein 78 (GRP78) in unstressed cells. Upon ERS, GRP78 is released and sequestered on unfolded proteins, allowing activation of PERK, IRE1-α, and ATF6.

**Figure 3.**
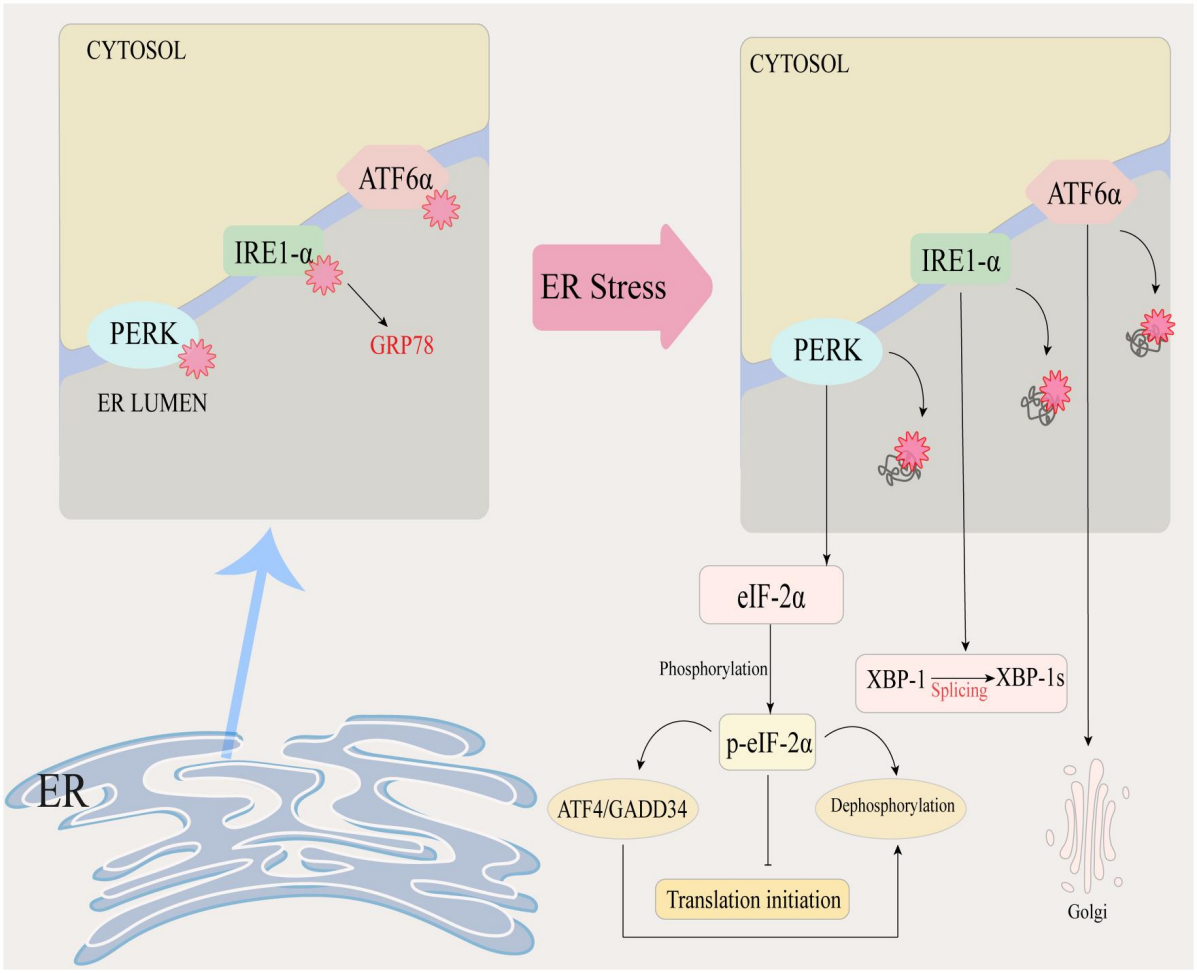
Mechanisms of endoplasmic reticulum stress initiation. The accumulation of unfolded proteins results in the release of GRP78, which activates PERK, IRE1-α, and ATF6. These proteins activate transcription and inhibit translation. GRP78 = glucose-regulated protein 78, PERK = PKR-like endoplasmic reticulum kinase, IRE1-α = inositol-requiring enzyme 1-α, ATF6 = activating transcription factor 6.

Activation of PERK results in the phosphorylation of eIF-2α, which not only regulates the translation process but also reduces the activity of related inflammatory factors and maintains redox balance [60]. Similarly, IRE1-α, in addition to its role in X-box binding protein 1 (XBP1) splicing, is involved in cellular signaling. Upon activation, IRE1-α can interact with the adaptor protein TNFR-associated factor 2 (TRAF2), leading to the activation of c-Jun N-terminal kinase (JNK) and NF-κB [68]. These interactions indicate that the IRE1-α branch of the UPR not only regulates adaptation to ERS and cell survival via XBP1 splicing but also activates signaling pathways involved in inflammation and apoptosis. Finally, ATF6α, after being cleaved and activated in the Golgi apparatus, translocates to the nucleus, where it mildly upregulates C/EBP Homologous Protein (CHOP), leading to autophagy [69]. Hepatic fibrosis is frequently associated with significant ERS [69]. Consequently, elucidating the molecular mechanisms of ERS upstream signaling represents a pivotal research avenue for targeted intervention in hepatic fibrosis [70, 71]. The initiation mechanism of ER stress is illustrated in Figure 3.

### Interaction between ADPN and ERS

Studies have shown that ADPN enhances lipid metabolism and reduces ERS via signaling pathways, such as XBP1, activating transcription factor 4 (ATF4), and acetyl-CoA carboxylase (ACC) [63]. ADPN exists in two circulating isoforms: globular adiponectin (gADPN) and full-length adiponectin (fADPN). AdipoR1 has a high affinity for gADPN, whereas AdipoR2 shows moderate affinity for both isoforms [10]. Furthermore, gADPN binds to AdipoR1 on macrophages, mediating the activation of NFκB and prompting the secretion of TNF-α and IL-6, contributing to the inflammatory response [72]. Conversely, fADPN stimulates the polarization of macrophages from M1 to M2 phenotype and fosters an anti-inflammatory state in macrophage signaling pathways via the AdipoR2-mediated AMPK pathway [73]. Additionally, ADPN regulates mitochondrial bioactivity [74] and maintains internal homeostasis via the AMPK pathway [44].

In hepatic tissues, AdipoR1 predominantly activates the AMPK pathway. Through this pathway, ADPN suppresses the expression of sterol regulatory element-binding protein 1c (SREBP1c), thereby reducing ERS [75] in hepatic lesions [76]. Activation of the PPARα pathway by AdipoR2 leads to increased phosphorylation of AMPK and p38 mitogen-activated protein kinase (MAPK) [77]. Activation of the PPARα pathway by AdipoR2 leads to increased phosphorylation of AMPK and p38MAPK. Binding of ADPN to AdipoR2 enhances PPARα ligand activity, promoting fatty acid oxidation, glucose utilization, and improved lipid metabolism, thereby reducing hepatic fibrosis [78]. These reactions are regulated by the phosphotyrosine-binding structural domain and leucine zipper motif (APPL1) [79, 80]. Furthermore, AdipoR1 and its associated proteins have been identified as crucial for ADPN’s anti-fibrotic effect in conjunction with APPL1. ADPN regulates APPL1 by facilitating the entry of extracellular calcium ions through AdipoR1, activating AMPK and sirtuin 1 (SIRT1). This, in turn, further stimulates the AMPK pathway and increases the expression of peroxisome proliferator-activated receptor gamma coactivator 1-alpha (PGC-1α) through deacetylation, in synergy with PPARα. Phosphorylation of PGC-1α enhances mitochondrial biogenesis and oxidative capacity, thereby slowing hepatic fibrosis progression [81].

**Figure 4.**
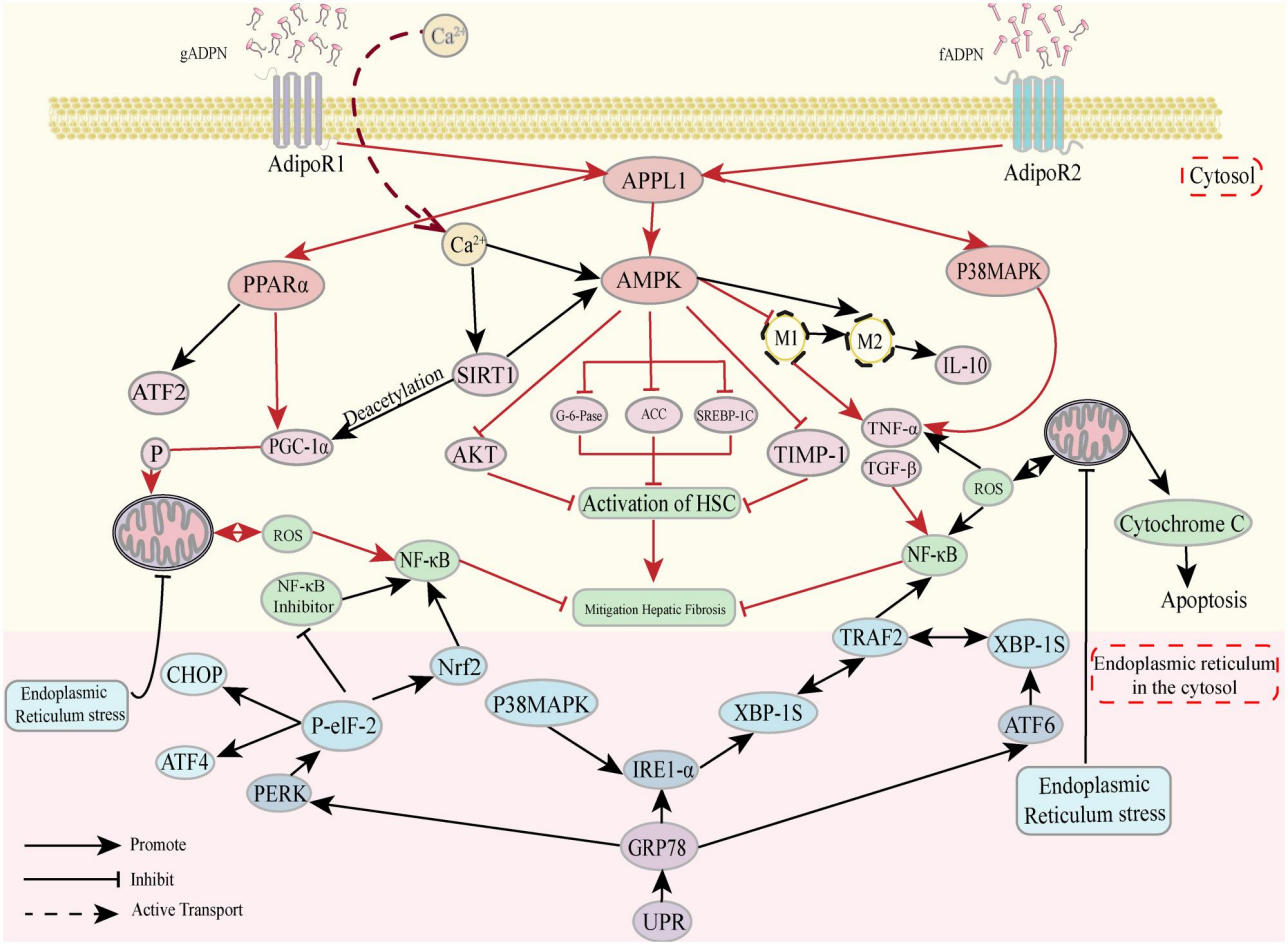
Mechanisms of ADPN to alleviate ERS and hepatic fibrosis. Relationship between downstream signaling of the ADPN and ERS. The red-labeled portion of the figure illustrates the principal pathway of action of ADPN on hepatic fibrosis. ADPN = adiponectin, ERS = endoplasmic reticulum stress.

ADPN suppresses pro-inflammatory factors, such as TNF-α by regulating macrophage polarization and enhances the expression of anti-inflammatory factors like IL-10. This process mitigates the UPR-induced activation of inflammatory pathways, including JNK and NFκB, thus slowing the progression of hepatic fibrosis. Furthermore, the ADPN-mediated p38 MAPK pathway directly influences IRE1-α during the UPR process, facilitating XBP1 splicing and the catabolism of the extracellular matrix. The specific pathways through which ADPN alleviates hepatic fibrosis are illustrated in Figure 4.

## Application of ADPN in the clinic

### ADPN serves as a biological risk marker for liver fibrosis

Hepatic fibrosis, a reversible pathological process, progresses to cirrhosis and ultimately end-stage liver disease in patients with chronic liver injury. The reversal and prevention of hepatic fibrosis have emerged as pivotal objectives in the clinical trials of novel anti-fibrotic therapies. Hepatic biopsy remains the gold standard for confirming fibrosis stages. However, this procedure carries risks, including sampling errors and complications. Additionally, there is a need to further explore non-invasive risk biomarkers for hepatic fibrosis in extensive research studies. ADPN, a promising biomarker, is characterized by high stability, low diurnal variation, and prevalent levels in the human body [82]. It has the potential to be a valuable tool for clinical studies, prognosis, and treatment of hepatic fibrosis in patients with chronic liver disease.

Currently, ADPN is known to primarily exert hepatoprotective and anti-fibrotic effects in liver injury contexts. Additionally, elevated circulating levels of ADPN correlate with an increased risk of developing hepatic fibrosis in individuals with chronic liver disease. Further investigation is needed to clarify the relationship between ADPN and other liver diseases [82, 83]. Numerous studies aim to investigate associations between ADPN concentrations and chronic liver fibrosis due to different liver diseases, including cirrhosis, biliary atresia, hepatitis C and B virus infections [84], non-alcoholic fatty liver disease, and insulin resistance. These study results are presented in Table 1. In a cross-sectional study of 232 patients with chronic liver disease, Balmer *et al.* [83] observed that serum ADPN levels were significantly elevated in patients with cirrhosis compared to patients without cirrhosis. Furthermore, serum ADPN levels demonstrated a positive correlation with elastography values (*P *<* *0.001), serum bile acids (*P *<* *0.001), and serum hyaluronic acid (*P *<* *0.001), which are surrogate biomarkers of liver fibrosis [83]. A related study has also confirmed the potential of ADPN as a biomarker for the risk of hepatic fibrotic lesions [85].

**Table 1.** Studies on circulating adiponectin (ADPN) levels in chronic liver disease

Year	Diagnosis	Study design	Population characteristics	Significant results
2009 [115]	Non-alcoholic fatty liver disease	Cross-sectional study	42 patients with non-alcoholic fatty liver disease	Adiponectin levels were independent predictors of advanced fibrosis
2022 [90]	Type 2 diabetes Non-alcoholic liver disease	Cross-sectional study	28 patients without alcoholic liver disease, 32 with hepatic steatosis alone, and 19 with alcoholic liver disease combined with significant hepatic fibrosis	A reduction in blood ADPN levels is a significant risk factor for the development of non-alcoholic liver disease in men with type 2 diabetes mellitus.
2011 [116]	Chronic hepatitis C	Case–control study	54 insulin-resistant and non-diabetic patients with chronic hepatitis C	In patients with chronic hepatitis C, fibrosis is associated with elevated adiponectin levels.
2010 [83]	Chronic liver disease	Cross-sectional study	64 patients with non-alcoholic fatty liver disease, 123 patients with other liver diseases, and 45 patients with cirrhosis.	In patients with cirrhosis, circulating ADPN levels are elevated. ADPN levels are positively correlated with the markers of hepatic fibrosis.
2013 [86]	Chronic hepatitis C	Case–control study	45 men with untreated chronic hepatitis C and 15 healthy men.	Serum ADPN levels were elevated in patients with hepatic fibrosis.
2018 [117]	Cirrhosis	Case–control study	122 patients with cirrhosis and 30 healthy controls	The levels of ADPN were higher in cirrhotic patients than in controls.
2011 [88]	Chronic hepatitis C	Retrospective study	325 patients with chronic hepatitis C	Elevated serum ADPN levels are an independent risk factor for future hepatocellular carcinoma in patients with chronic hepatitis C.
2008 [87]	Insulin resistance Chronic hepatitis C	Prospective study	152 patients with chronic hepatitis C	ADPN levels are significantly associated with insulin resistance.
2012 [118]	Insulin resistance Chronic hepatitis C	Multicenter Study	103 treated with ribavirin and pegylated interferon-α2a in patients with chronic hepatitis C	A negative correlation was observed between hepatic steatosis and ADPN.
2013 [89]	Insulin resistance Chronic hepatitis C	Case–control study	40 patients with hepatitis C and 40 healthy controls	A significant reduction in ADPN levels was observed in patients with hepatitis C, with a corresponding increase in insulin resistance.
2008 [84]	Chronic hepatitis C	Case–control study	83 patients with chronic hepatitis C, 59 patients with chronic hepatitis B, and 43 healthy controls.	Serum ADPN is an independent predictor of hepatic steatosis and treatment prognosis.
2011 [85]	Chronic hepatitis C	Case–control study	97 patients with hepatocellular carcinoma and 97 healthy controls.	Serum ADPN levels have been demonstrated to be a predictor of liver fibrosis.

Furthermore, a study conducted by Korah *et al.* [86] revealed that ADPN serum levels are elevated in patients diagnosed with hepatic fibrosis due to hepatitis C, substantiating the specificity of ADPN in this context. Further studies have demonstrated that ADPN is also associated with liver cancer and insulin resistance in patients with hepatitis C [87–89]. Additionally, ADPN is strongly linked to the development of non-alcoholic liver disease in diabetic patients [90].

### Detection methods for ADPN

While liver biopsy remains the definitive standard for staging hepatic fibrosis, the use of highly efficient and specific non-invasive detection methods is crucial for the real-time monitoring of disease development and progression. ADPN is emerging as a promising non-invasive biochemical marker for assessing the severity of hepatic fibrosis [82]. Initially, detection of ADPN primarily relied on radioimmunoassay (RIA) or RIA-based methods. However, the RIA method is costly, involves lengthy and environmentally detrimental sample processing, and poses challenges to full automation.

Latex particle-enhanced turbidimetric immunoassay (LTIA) and enzyme-linked immunosorbent assay (ELISA) currently stand as the methods of choice for ADPN detection in clinical laboratories. The study demonstrated a statistically significant positive correlation between serum ADPN results detected by ELISA and LTIA, and the regression equation was *y* (LTIA) = 0.953*x* (ELISA) + 0.39 (*r *=* *0.990) [91]. LTIA is extensively employed for the quantitative detection of ADPN across diverse biological samples, attributed to its straightforward raw material production and heightened analytical sensitivity [91]. Compared to alternative marker analysis techniques, LTIA offers simplicity, rapidity, and superior accuracy, particularly for detecting low concentrations of ADPN.

### Clinical therapeutic directions for ADPN

#### Targeted therapies for ADPN

Recent studies increasingly show that ADPN protects the liver primarily by inhibiting the proliferation, migration, and activation of HSCs and myofibroblasts, key factors in hepatic fibrosis development. Consequently, enhancing the circulation of ADPN or utilizing ADPN receptor agonists may represent a promising avenue for the protection and treatment of hepatic fibrosis. Currently, two main methods exist for increasing ADPN concentration *in vivo*.

The first approach involves exogenously administering ADPN via an adenoviral vector, exemplified by ADP355. This method is used in preclinical models. ADP355, a synthetic peptide mimicking ADPN, effectively reduces symptoms of carbon tetrachloride-induced hepatic fibrosis in mice. Furthermore, ADP355 has shown anti-fibrotic effects, reduced expression of pro-fibrotic markers like α-SMA, TGF-β1, and TIMP-1, suggesting potential therapeutic applications [79]. ADP355 also promotes AMPK phosphorylation. Given these findings, ADP355 could be a promising new anti-fibrotic agent for liver fibrosis treatment. However, the lack of clinical data on hepatotoxicity requires further evaluation of ADP355 to confirm its efficacy and clinical utility.

An alternative approach is the exogenous supplementation of substances that promote the secretion or expression of ADPN. Recent evidence shows that activating PPAR-γ increases ADPN expression and circulation through gene transcription regulation [92]. Consequently, the PPAR-γ cascade response modifies the direct targets for potential clinical applications of ADPN. Currently, thiazolidinediones (TZDs), clinically used PPAR-γ agonists, exhibit antiproliferative and anti-inflammatory effects by activating PPAR-γ. They also promote the release of ADPN from adipose tissue into the bloodstream. However, TZDs are extensively used to enhance insulin sensitivity in diabetic patients [92]. Further investigation is required to elucidate the mechanisms by which ADPN regulates hepatic fibrosis. Therefore, identifying new pharmaceutical agents targeting ADPN for treating hepatic fibrosis is crucial for medical breakthroughs and innovations.

Presently, clinical trials are evaluating a range of pharmacological agents targeting diverse pathways for the treatment of liver fibrosis in animal models. However, these pharmacological agents are associated with significant adverse effects. For example, the use of PPAR agonists markedly elevates the risk of cardiovascular events, fractures, and bladder cancer in patients [93]. To date, no pharmacological treatments for hepatofibrosis have received approval. ADPN mitigates hepatic insulin resistance, facilitates fatty acid oxidation and glucose metabolism, diminishes hepatic inflammation and the progression of fibrosis, and serves as an indicator of hepatic fibrosis severity [94, 95]. As previously described, ADPN/AdipoR2 signaling initiates the hepatic PPARα pathway and suppresses pro-inflammatory gene expression, while ADPN/AdipoR1 signaling engages the hepatocellular AKT pathway to modulate insulin resistance, cell proliferation, and apoptosis. Specifically, ADPN administration leads to a reduction in ceramide levels, enhanced hepatic insulin sensitivity in the liver and other tissues, and a reduction in hepatic inflammation [96]. Numerous investigations have validated the ADPN-AdipoR system as a potential therapeutic target for liver fibrosis. Recently, the dual AdipoR1/AdipoR2-based agonist JT003 has been demonstrated to enhance fatty acid oxidation and glucose uptake and mitigate liver fibrosis and insulin resistance in mice via signaling pathways, including AMPK, PPARα, and AKT [44].

Furthermore, a recent study has illustrated the role of ADPN in inhibiting foam cell formation to prevent cardiovascular diseases [97], exhibiting anti-inflammatory effects in chronic lung diseases [98], influencing psoriasis development [99], and potentially being linked to the onset of Alzheimer’s disease [100].

#### Combination therapy of ADPN with anti-insulin resistance drugs

The liver plays a pivotal role in regulating insulin resistance. The results of numerous studies have demonstrated that insulin resistance originates in the liver and subsequently develops in skeletal muscle. In recent years, adipokines have also been identified as another crucial target of insulin. As an adipokine, ADPN has been demonstrated to be a pivotal modulator of insulin sensitivity in animal experiments.

Recently, fibroblast growth factor-21 (FGF21) was demonstrated to be a potent insulin sensitizer in an insulin-deficient primate model [101]. The liver was identified as the major FGF21 expression site, while adipose tissue was identified as the target tissue. The administration of exogenous FGF21 was found to significantly improve glucose utilization in animals. Moreover, it was demonstrated that the acute hypoglycemic activity of FGF21 was not only mediated by ADPN [102] but also served as a potent regulator of ADPN secretion. Additionally, FGF21 was heavily dependent on ADPN for its insulin-sensitizing effects [103]. The result demonstrated that FGF21 is capable of activating the AMPK pathway in both *in vivo* and *in vitro* models, thereby regulating energy homeostasis. Furthermore, these energetic changes were found to have an integral impact on insulin sensitivity [104]. Further studies have demonstrated that it is possible to attenuate the associated liver injury through the FGF21/ADPN pathway [105, 106]. Consequently, the potential of a combination of ADPN and FGF21 for the treatment of liver fibrosis and related metabolic syndromes is an area worthy of further investigation.

The degree of insulin sensitivity is contingent upon the binding of insulin to its receptor, which is subsequently phosphorylated and initiates downstream signaling cascades. It has previously been demonstrated that PPARγ is involved in the regulation of ADPN expression and secretion, insulin sensitivity, and the alleviation of symptoms associated with insulin resistance [107]. Furthermore, the study demonstrated that PPARγ agonists also elevate serum ADPN levels, and that the insulin-sensitizing impact of PPARγ agonists is partially dependent on ADPN [108]. In an animal experiment on Olmesartan [109], it was demonstrated that Olmesartan inhibits the expression of AGE/RAGE/p-JNK pathways while increasing the PPARγ/ADPN pathway, thereby alleviating the symptoms of liver injury in patients with type 2 diabetes mellitus. Consequently, insulin sensitization combined with anti-hepatic fibrosis therapy targeting PPARγ/ADPN represents a potential emerging therapy.

**Figure 5.**
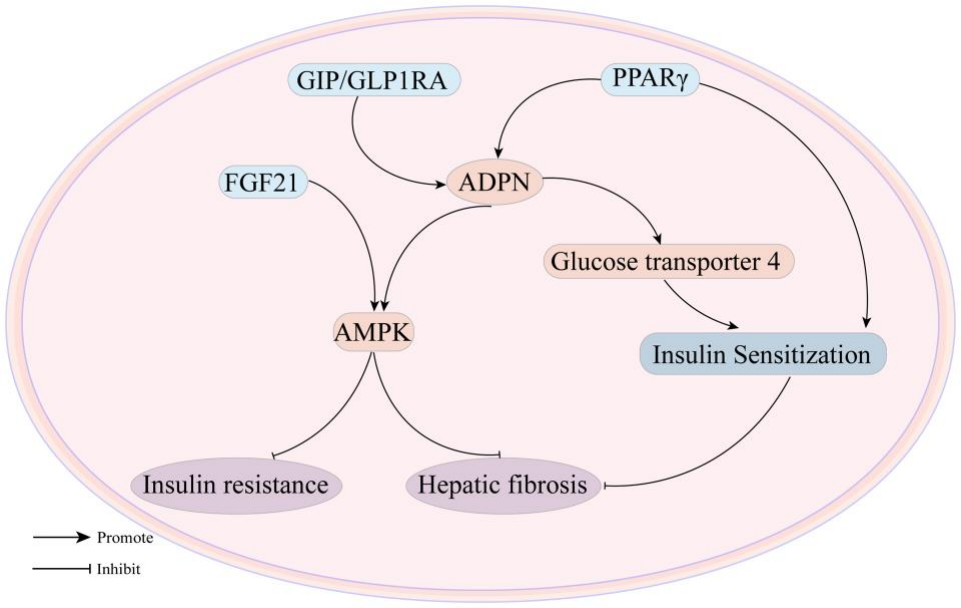
ADPN clinical therapies. A mechanistic sketch of ADPN in combination therapy with anti-insulin resistance drugs. ADPN = adiponectin.

The potential insulin sensitization mechanisms of ADPN are primarily increased glucose transporter 4-mediated glucose uptake in white adipocytes, decreased visceral fat storage, and decreased hepatic lipid accumulation [110]. Furthermore, ADPN levels have been demonstrated to be negatively correlated with insulin resistance in rodent models of obesity and in multiple human cohorts [111]. A study has demonstrated that tirzepatide, a glucagon-promoting polypeptide (GIP) and glucagon-like peptide-1 (GLP-1) activator, can increase ADPN levels [112, 113]. Moreover, tirzepatide has been demonstrated to reduce levels of non-alcoholic steatohepatitis-associated biomarkers in diabetic patients in a cohort study of type 2 diabetes mellitus [114]. Therefore, modulation of ADPN levels by GIP/GLP1RA activators not only improves tissue insulin sensitivity but also reduces associated liver disease symptoms. The mechanism of action of the combination therapy of ADPN and insulin resistance is illustrated in Figure 5.

## Conclusion

ADPN, a multifunctional adipokine, circulates at high levels. Its levels predict the progression of several diseases, including obesity, diabetes, atherosclerosis, and chronic liver diseases, such as Hepatitis C Virus (HCV), Hepatitis B Virus (HBV), and cirrhosis. ADPN exhibits hepatoprotective properties, enhancing hepatocyte viability, increasing hepatic insulin sensitivity, and reducing liver inflammation and fibrosis. Furthermore, serum ADPN levels correlate with hepatic fibrosis severity. This review explores the correlation between hepatic fibrosis and serum ADPN levels and elucidates how ADPN affects aHSC proliferation, migration, and apoptosis. Additionally, it outlines ADPN’s role in curbing hepatic fibrosis progression by mitigating endoplasmic reticulum and mitochondrial dysfunction through downstream signaling pathways. It also highlights that ADPN pharmacotherapy significantly reduces hepatic fibrosis progression in *in vitro* studies and animal models. Additionally, advances in ADPN detection technology position it as a promising new therapeutic and biomarker for liver fibrosis, assessing both disease risk and severity.

## Authors’ Contributions

T.C. and J.X. were involved in the study design and supervision. T.C. and W.Y. drafted the initial manuscript. R.D., H.Y, and Q.Z. revised the manuscript. M.S. and J.W. were involved in the acquisition and interpretation of data. All authors read and approved the final manuscript.

## Data Availability

Not applicable.
